# A study of medical professionals’ perspectives on medicines containing codeine in South Africa

**DOI:** 10.4102/sajpsychiatry.v24.i0.1162

**Published:** 2018-06-27

**Authors:** Michelle Foley, Tara Carney, Eileen Rich, Siphokazi Dada, Chrisitne Mburu, Charles Parry

**Affiliations:** 1 School of Health Sciences, Waterford Institute of Technology, Ireland; 2 Alcohol, Tobacco and Other Drug Research Unit, South African Medical Research Council, South Africa; 3 The Local Choice, Cur-o-Pharm Pharmacy, South Africa; 4 South African Medical Research Council, South Africa

## Abstract

**Background:**

Misuse of codeine available on prescription and over-the-counter (OTC) has been highlighted as a potential problem in South Africa.

**Objective:**

To examine the perceptions of medical professionals regarding codeine use (prescribed and OTC), misuse, dependence and treatment options in South Africa.

**Method:**

Data for the study were obtained using a sample of medical professionals obtained through random and convenience sampling. A quantitative methodology was employed using a structured self-administered questionnaire with closed and open-ended items. Data analysis was conducted using SPSS version 21; 238 medical professionals involved in the prescribing of codeine completed the questionnaire.

**Results:**

Two-thirds of participants stated that they routinely reviewed patients prescribed codeine, and high levels of concern were expressed about the availability of OTC medicine containing codeine in pharmacies (84.9%) and on the internet (71.3%). There was agreement that medicines containing codeine should be regulated to a prescription-only medicine (85.3%). Only 22% of participants agreed that they had suitable screening methods to help with detection of codeine dependence. Eighty per cent indicated that they would welcome the opportunity for greater instruction on prescribing potentially addictive medicines.

**Conclusion:**

There appears to be a need to improve education on consumption and risks associated with codeine use. In addition, screening tools are needed to detect those with codeine dependence. Greater data sources are now needed to examine the sale of and consumption of codeine medicines in the interest of public health.

## Introduction

Opioids are widely used in the management of pain-related conditions in primary health care. Codeine, a weak opioid, is commonly prescribed for the acute onset of pain, as a treatment for cough and to a lesser extent for diarrhoea.^1^ In recent times, misuse of codeine medicines has been highlighted as a potential problem in South Africa.^2,3^ Misuse of codeine is defined as using medication obtained with or without a medical prescription outside of accepted medical guidance. This may include dosage regimes that are higher or used for longer periods than medically advised, for the purpose of intoxication, including recreational use, or where the risks outweigh the benefits.^4^ Dependence is classified as a psychiatric disorder under the DSM-IV diagnostic criteria.^5^

One of the principal factors considered to contribute to problematic codeine use is its availability without a medical prescription.^6^ In South Africa, codeine combination products containing codeine combined with paracetamol, ibuprofen or aspirin are sold without a medical prescription under the supervision of a pharmacist at unit doses of up to 20 mg of active ingredient. Codeine linctus is also sold over-the-counter (OTC) and contains up to 10 mg of codeine phosphate per 5 mL. Significant health problems including gastrointestinal haemorrhage, nephrotoxicity, hypokalaemia and acute haemorrhagic necrotising pancreatitis have been reported from continued codeine use, attributed to the combined drug components (paracetamol, ibuprofen or aspirin) being used in higher doses than recommended.^6^ In recent times, some national-based initiatives have been developed aimed at reducing the risk associated with codeine consumption.^7^ One such initiative, the Codeine Care project, rolled out by the self-manufactures association of South Africa, is designed to monitor purchases and help identify individuals who may be misusing or be dependent on codeine.^8^ The Medical Control Council has also responded to the potential risk associated with OTC codeine consumption and is considering changes to the current regulation on OTC codeine sales, reducing the permissible amount of codeine per unit dose from 20 mg to 10 mg of active ingredient.^9^ Additional factors integral to developing a codeine problem appear to be a lack of awareness concerning risks associated with OTC and prescription drug use.^10^ Generally, OTC and prescription medications are not perceived to be as harmful as illicit drugs and are widely available and accepted by the general community.

There is limited data on levels of prescribing of codeine, consumption levels and prevalence rates of codeine use, misuse and dependence in South Africa. Medical professional perspectives surrounding prescribed and OTC codeine medicine use are scant. There is also inadequate information on how medical professionals screen, detect or treat codeine misuse in primary care. Many general practitioners (GPs) are thought to remain unaware of patients’ self-medication habits and appear to find difficulties in identifying codeine dependence without first being informed by the patient. The perspectives of medical professionals are highly relevant to the current debate on codeine and, in particular, opinion on the continued OTC sale of the drug.

The aim of the study was to examine medical professionals’ perspectives on prescribed and OTC codeine, codeine dependence, screening and treatment options in South Africa. This study was conducted as part of a larger European Union (EU)-funded research project (www.codemisused.org) that aimed to quantify and explore prescribed, OTC and web-retailed codeine use, misuse and dependence in Ireland, United Kingdom and South Africa. Data for other countries are reported separately: (http://bmjopen.bmj.com/content/6/7/e011725; https://link.springer.com/article/10.1007/s11845-016-1546-z).

## Methods

A cross-sectional postal survey using a representative sample of prescribing practitioners in South Africa was conducted in 2014. The questionnaire was designed on a 5-point Likert scale, with several options for free text insertion (see attached questionnaire). Questions were created following a comprehensive review of the literature and following consultation with medical professionals and piloting of the questionnaire.^11^ Eight questions were used to determine the demographics of the sample. A sample size calculation indicated that to achieve an 80% power to detect a 50% difference in response distribution and a significance level of 0.05, 335 responses would be required. In order to gain representation from each professional group involved in the prescribing of codeine, selection for inclusion to the study was made proportionate to the number of professionals registered in South Africa. Based on the predicted response rate of 25%, the numbers were increased to ensure that the sample size was achieved. A random sample calculator was used to generate a random numbers list and the corresponding details were extracted from the directory and prepared for mailing by three members of the research team. Professionals were chosen proportionate to the numbers working in each area (GP, family medicine, pain specialist). One thousand GPs, 241 family medicine practitioners (FMPs) and 214 specialist practitioners (SPs) were mailed the questionnaire with a covering letter requesting participation, an information sheet and a consent form. A stamped addressed return envelope was included. A postcard reminder followed 3 weeks later. Each participant was also given the opportunity to complete the questionnaire online (Bristol online surveys) and a link to facilitate this was provided in the covering letter.

This recruitment phase resulted in a total of 46 responses. Ten questionnaires were returned indicating that the GP was retired, 6 were returned as ‘deceased’, 10 had moved to another practice or emigrated, 53 post boxes were closed and a further 26 were returned as ‘address unknown’. Issues with a postal strike resulted in an alternative recruitment strategy in order to reach the desired sample. A random sample of 1000 e-mail addresses was purchased from an independent marketing company from their list of 5500 private practitioners. These names were checked against the original sample to ensure no duplication had occurred. This group was e-mailed and asked to participate in the questionnaire by following the attached link. This resulted in the recruitment of a further 70 GPs. As the desired target sample was not reached, an incentive (R200) was added, and a further 2000 e-mail address were purchased, checked for duplication and e-mailed the questionnaire link as previously described. This resulted in the recruitment of a further 94 participants. Twenty GPs were recruited as a result of face-to-face contact at a medical conference. In total, 238 participants were recruited to the study.

Data analysis was conducted using SPSS version 21. Data were coded by defining and labelling each of the variables and assigning numbers to each of the possible responses. This was then entered manually and combined with data extracted from the questionnaires captured online. Data were screened and checked for errors by checking for maximum and minimum values and using case summaries. Descriptive statistics were used to describe the data. Data from the open-ended questions were entered into an Excel spreadsheet. Quotes were entered verbatim and combined with content obtained from the online questions. Content was examined in each question and then coded by one researcher into broad themes. The themes were discussed by two members of the research team and categories were applied for subsequent coding. Four researchers independently coded the data (2 academics and 2 pharmacists). Coder reliability of the data was conducted by dialogue between four members of the research team. Each item was checked for both agreement and non-agreement with the thematic categories. Where discrepancies or non-agreement occurred, the researchers discussed the illustrated content. This was then resolved when two or more researchers were in agreement. Data were then compiled to show the frequency of categorical items as a percentage of those responding to each of the open-ended questions. The suspected occurrence of dependence problems was calculated as a rate using the number of consultations indicated by the respondents. Referrals were calculated and presented as an overall percentage of participants’ referrals to secondary care. The research was approved by the South African Medical Research Ethics Committee in 2014.

## Ethical consideration

The research was approved by the South African Medical Research Ethics Committee in 2014.

## Results

Table 1 shows the demographic details of the participants. The mean age of participants was 54 years (range 30–89), and the average years of practice was 28.31 years (range 3–58).

**TABLE 1:** Demographic information of study respondents.

Variable	Frequency (*f*)	%
**Gender**		
Male	163	68.8
Female	74	31.2
**Profession**		
General practitioner	184	77.3
Specialist in family medicine	14	5.9
Specialist in pain management	19	8
Other	21	8.8
**Location**		
Urban	176	74.3
Rural	24	10.1
Mix of both	37	15.6
**Specialist training in substance misuse**		
Yes	11	4.7
No	224	95.3

*N* = 238.

Table 2 shows the results for statement items related to medical practitioners’ experiences of prescribing codeine. The majority of participants routinely review patients prescribed codeine (i.e. strongly agreed or agreed). About one-third of participants had experienced some degree of resentment towards them when they questioned patients about their codeine use, while a slightly higher level indicated the opposite (46%). However, only a very small minority of respondents felt awkward about asking patients about their codeine use (15%). Just over one-third of participants thought requests for prescribed codeine were on the increase with similar levels disagreeing. The principals of good prescribing appeared in place with over three-quarters of the total sample avoiding the prescribing of codeine with other drugs producing a depressant effect on the central nervous system. Similarly, non-opioid analgesics appeared to be the first line of treatment with 68% of participants stating that they prescribed opioid analgesics following initial unsuccessful pain relief with non-opioid treatments. However, codeine linctus was more readily prescribed as a first line of treatment for cough. There were mixed views regarding the efficacy of codeine in doses of < 30 mg. Thirty-four per cent of participants thought that doses of < 30 mg of codeine were not effective in the treatment of mild to moderate pain, while 42% felt the opposite. One-quarter of the total sample indicated a neutral response for this question.

**TABLE 2:** Level of agreement, disagreement and neutral responses with each of the statements related to codeine use, including prescribed codeine.

Statement	Strongly agree	Agree	Neutral	Disagree	Strongly disagree
	*f* (%)	*f* (%)	*f* (%)	*f* (%)	*f* (%)
I routinely review patients who are prescribed medicines containing codeine	56 (23.5)	101 (42.4)	34 (14.3)	40 (16.8)	7 (2.9)
I believe that patients resent me asking about their use of medicines containing codeine	15 (6.3)	57 (23.9)	56 (23.5)	88 (37.0)	21 (8.8)
I feel awkward about asking patients about their codeine use because they will think I am accusing them of having a problem	3 (1.3)	25 (10.5)	34 (14.3)	115 (48.3)	61 (25.6)
Patients are aware of adverse health consequence associated with high doses of combination codeine preparations	4 (1.7)	41 (17.2)	35 (14.7)	108 (45.4)	50 (21.0)
It is unlikely that prescribed medicines containing codeine are used as recreational drugs	12 (5.0)	40 (16.8)	40 (16.8)	94 (39.5)	49 (20.6)
Patients’ requests for prescribed medicines containing codeine is increasing	20 (8.4)	70 (29.4)	67 (28.2)	72 (30.3)	9 (3.8)
I would avoid prescribing medicines containing codeine with other drug groups that also produce a depressant effect on the central nervous system	66 (27.7)	115 (48.3)	37 (15.5)	18 (7.6)	2 (0.8)
I would generally prescribe medicines containing codeine following unsuccessful treatment with non-opioid analgesics	29 (12.2)	132 (55.5)	27 (11.3)	37 (15.5)	10 (4.2)
I would generally prescribe codeine linctus following unsuccessful treatment of cough with non-codeine containing cough suppressants	14 (5.9)	75 (31.5)	32 (13.4)	72 (30.3)	45 (18.9)
Doses of < 30 mg of codeine phosphate (compounded or uncompounded) are not very effective for treating mild to moderate pain	19 (8.0)	61 (25.60)	58 (24.4)	82 (34.5)	17 (7.1)

Table 3 illustrates the responses to questions regarding OTC codeine. There appeared to be a good professional practice with regard to enquiry around patients’ self-medicating behaviours, with a high proportion of participants (66.0%) agreeing that they routinely asked patients about their use of OTC medicines (strongly agreed or agreed) and documented OTC codeine use in patients’ medical notes (64.0%). High levels of concern were expressed relating to the availability of OTC medicine containing codeine in pharmacies (84.9%) and on the internet (71.3%), and many felt that this medicine should be regulated to prescription only (85.3%). Overall, participants did not believe that OTC codeine gave patients better choice for pain relief or cough relief, and less than one-quarter of participants felt that codeine was more effective than non-opioid alternatives. Generally, participants thought that patients did not understand the risks of dependence when taking OTC codeine medication (95.0%), and a large proportion agreed that patients believed OTC medicines containing codeine were safe (89.2%). Overall, participants did not think that patients were given enough information on OTC medicine use and believed that they could easily use them as recreational drugs and felt codeine misuse was a serious problem to society. The ability to extract codeine from combination formulation appeared not to be widely known by health professionals.

**TABLE 3:** Level of agreement, disagreement and neutral responses with each of the statements related to over-the-counter codeine.

Statement	Agree strongly	Agree	Neutral	Disagree	Disagree strongly
	*f* (%)	*f* (%)	*f* (%)	*f* (%)	*f* (%)
I routinely ask patients about their use of over-the-counter medicines	42 (17.6)	115 (48.7)	32 (13.4)	42 (17.6)	5 (2.1)
I document the use of over-the-counter medicine in a patient’s medical notes	36 (15.1)	117 (49.2)	42 (17.7)	37 (15.6)	5(2.1)
I am concerned about the availability of over-the-counter medicines containing codeine in pharmacies	93 (39.1)	108 (45.8)	23 (9.7)	12 (5.1)	0 (0.0)
The availability of medicines containing codeine on the internet is a growing concern for the medical profession	71 (30.3)	96 (41.0)	59 (25.2)	7 (3.0)	1 (0.4)
The potential to buy medicines containing codeine from multiple sources adds significantly to the potential for misuse	129 (54.7)	92 (39.0)	11 (4.7)	3 (1.3)	1 (0.4)
Patients are given sufficient information on use of over-the-counter medicines containing codeine	6 (2.5)	14 (5.9)	23 (9.7)	118 (49.8)	76 (32.1)
Medicines containing codeine should be regulated to a prescription-only medicine (POM)	123 (51.7)	80 (33.6)	16 (6.7)	12 (5.1)	6 (2.5)
Over-the-counter medicines containing codeine give patients better choice for pain relief	6 (2.5)	41 (17.4)	50 (21.2)	92 (39.0)	47 (19.9)
Over-the-counter mixtures containing codeine give patients better choice for treating cough	2 (0.9)	31 (13.2)	42 (17.6)	95 (40.4)	65 (27.7)
Over-the-counter medicines containing codeine are more effective than non-opioid analgesics such as paracetamol and ibuprofen in treating mild to moderate pain	4 (1.7)	44 (18.6)	42 (17.7)	107 (45.1)	40 (16.9)
The potential for misuse of over-the-counter medicines containing codeine is minimal	4 (1.7)	9 (3.8)	11 (4.6)	124 (52.3)	89 (37.6)
Over-the-counter medicines containing codeine have greater potential for inappropriate use compared to prescribed medicines containing codeine	64 (27.0)	131 (55.0)	16 (6.8)	15 (6.3)	11 (4.6)
Codeine is easily extracted from compounded formulations (e.g. Cocodamol) increasing its abuse potential	13 (5.5)	62 (26.1)	145 (61.7)	11 (4.6)	4 (1.7)
It is likely that over-the-counter codeine medicines could be used as recreational drugs	39 (16.4)	140 (59.3)	40 (16.8)	15 (6.3)	2 (0.8)
Codeine misuse is as serious a problem to society as misuse of stronger opioids	58 (24.4)	117 (49.4)	40 (16.9)	19 (8.0)	3 (1.3)
Patients do not fully understand the risk of dependence in taking over-the-counter medicines containing codeine	94 (40.0)	126 (53.6)	7 (2.9)	5 (2.1)	3 (1.3)
Patients believe that over-the-counter medicines containing codeine are safe	76 (32.1)	136 (57.1)	17 (7.1)	7 (3.0)	1 (0.4)

Table 4 depicts the responses of participants in relation to codeine dependence, options for screening and treatment. There was mixed agreement as to whether patients were not at risk of developing codeine dependence if they took their medication as prescribed with 38% agreeing and 45% disagreeing. Almost half (45%) of respondents indicated that they found it difficult to identify problematic use of medicines containing codeine without the patient first informing them and less than a third were confident in the identification of codeine dependence. Only one in five participants agreed that suitable screening methods were available to help them with detection and a similar proportion felt that there were adequate support services in their area to deal with codeine dependence. Most (80%) participants concurred that they would welcome the opportunity for greater instruction on prescribing potentially addictive medicine.

**TABLE 4:** Level of agreement, disagreement and neutral responses with statement items related to codeine dependence and treatment.

Statement	Strongly agree	Agree	Neutral	Disagree	Strongly disagree
	*f* (%)	*f* (%)	*f* (%)	*f* (%)	*f* (%)
Patients who take their codeine medication as prescribed are not at risk of developing a codeine dependence	7 (3.0)	83 (35.0)	41 (17.3)	92 (38.8)	14 (5.9)
Patients do not fully understand the risk of dependence when taking prescribed medicines containing codeine	41 (17.4)	164 (69.5)	17 (7.2)	13 (5.5)	1 (0.4)
I find it difficult to identify problematic use of medicines containing codeine (including OTC) without the patient first telling me	14 (5.9)	93 (39.2)	46 (19.4)	73 (30.8)	11 (4.6)
I am confident that I can identify codeine dependence in my patients	11 (4.6)	62 (26.2)	78 (32.9)	79 (33.3)	7 (3.0)
Females are at higher risk of developing a codeine dependence than their male counterparts	21 (8.9)	105 (44.3)	105 (44.3)	10 (4.2)	1 (0.4)
Codeine dependence can be managed effectively in general practice	6 (2.5)	76 (32.1)	66 (27.8)	81 (34.2)	8 (3.4)
I have suitable screening methods that I use to identify inappropriate use of medicines containing codeine	4 (1.7)	48 (20.3)	60 (25.3)	109 (46.0)	16 (6.8)
Support services are readily available in my area to help those with a codeine dependence problem	2 (0.8)	51 (21.5)	38 (16.0)	103 (43.5)	43 (18.1)
I am fully aware of best practice in managing codeine misuse and dependence	12 (5.1)	55 (23.3)	61 (25.8)	90 (38.1)	18 (7.6)
I would like more instruction on prescribing potentially addictive medications	60 (25.4)	126 (53.4)	34 (14.4)	14 (5.9)	2 (0.8)

The median number of patients suspected of being codeine dependent per 100 patient consultations was three patients per month. Of the respondents, 13% did not suspect any cases of codeine dependence in their patients and 43% of participants had made at least one referral to addiction services in the previous month.

**FIGURE 1:**
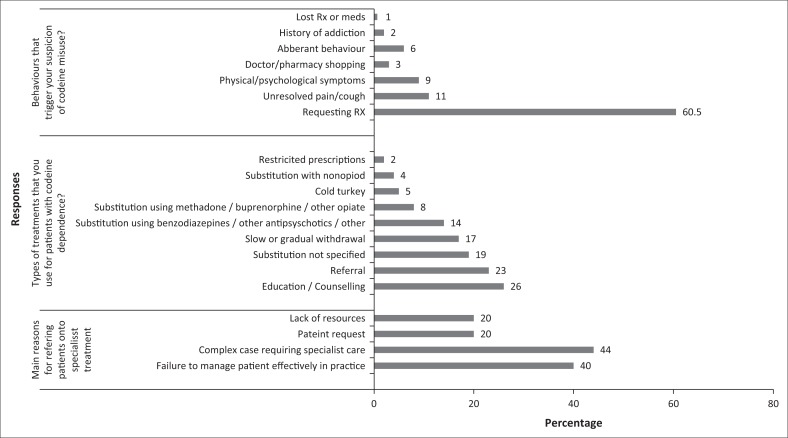
The responses in each thematic category as an overall percentage.

Patient behaviours triggering medical practitioners’ suspicion of codeine misuse are illustrated in Figure 1. The most likely indicator cited was requesting a prescription for codeine, and included situations such as asking for specific codeine brand names or requesting prescriptions for early refills. Complaining of unresolved pain was the second most likely indicator (11%) followed by the display of physical or psychological symptoms (9%). The most frequent type of treatment utilised was education and counselling techniques (26%) followed by substitution with another drug. Responses also indicated that slow and gradual withdrawal of the medication was used (17%). The primary reason for referring a patient to specialist care was the inability to effectively manage the patient in primary care (40%) or the case was complex and required specialised care (44%). Additional comments related to codeine use, misuse and dependence were expressed by participants in the open-ended questions. Some respondents spoke of codeine as a societal problem requiring attention and emphasised the lack of patient understanding regarding codeine consumption. Issues regarding the sale of OTC codeine in pharmacies and the requirement for improvement in the monitoring of sales were common threads.

## Discussion

The findings indicate that medical prescribers do ask patients about prescribed and OTC codeine use. Substantial concern was expressed by medical practitioners about the sale and availability of OTC codeine in pharmacies in South Africa. The concern relating to OTC is also evident amongst professionals in the UK.^12^ South Africa permits the sale of up to 20 mg of codeine phosphate for solid preparations and codeine cough linctus is widely available as an OTC medicine which is higher per unit dose than sold in most countries in the EU. Codeine linctus preparations contain up to 10 mg of codeine phosphate per 5 mL. Consuming codeine linctus limits the risk of toxicity often found with combination codeine preparations containing paracetamol and ibuprofen.^13^ Although efforts have been made to improve the regulation of OTC codeine in South Africa in recent years, including the introduction of patient registers in pharmacies, the majority of prescribers would like the medicine to be regulated to prescription only. While there is no direct evidence to suggest that OTC codeine is responsible for a large proportion of misuse and dependence issues, several countries across Europe, Australia and the USA have opted to impose greater restrictions on OTC codeine sales or alternatively not to dispense codeine without a medical prescription.^14,15^

This study found that the prescribing of codeine follows the principal of good prescribing practice, with a high proportion of medical professionals agreeing that codeine was only prescribed in response to unresolved pain or cough already treated with alterative non-opioid medicines. However, views regarding the effectiveness of codeine in treating pain in doses of < 30 mg were mixed, and this signals the complexity of treating pain-related conditions. The efficacy of low doses of codeine is not well documented in the literature, and the majority of studies are confined to postoperative acute pain at high doses.^16^ Further studies are required to examine the effectiveness of codeine found in many OTC medicines and where codeine is given over longer and extended periods.

There was a high level of agreement in this study that patients do not fully understand the risks of taking medicines containing codeine and believe them to be safe. Similar findings have been found in other studies examining patient understanding of medicine use and have highlighted the lack of patient understanding regarding drug terminology, knowledge of side effects and correct dosing regimens.^17,18^ Further research is also needed to establish patient knowledge and understanding of risks when taking medicines containing codeine and equally how the medical profession, including pharmacy staff, can heighten awareness of risk through communication and education techniques. It is clear that screening tools for detecting codeine dependence specifically are lacking in practice, with professionals relying on patient behaviours to identify potential misuse and dependence issues.^19^

Previous research has shown that there is a low level of treatment demand for specialist care.^2^ However, nearly half of all respondents in this study treating codeine-dependent patients indicated that they had made at least one referral to specialist care and agreed that treatment services were not readily available. In addition, it is feasible that a significant proportion of codeine dependence is managed through home detoxification and therefore remains under-reported. The treatment of codeine dependence and support for medical professionals in practice requires review in the context of the development of adequate services for patients within primary care, community and inpatient setting^18^ and the fact that few practitioners (less than 5%) reported that they had received specialist training in substance misuse.

There are several limitations with respect to this study. The recruitment of participants was challenging, and changes to recruitment procedure were necessary to improve the response rate. Therefore, participants were predominantly those practicing on the private register in South Africa, and thus, this study is likely to have responder bias. The findings are not generalisable to all medical practitioners in the country given the low rate of responses to the initial postal survey of medical practitioners on the HPCSA register. In spite of all efforts to recruit medical professionals, we only achieved 67% of the actual target number needed to adequately power the study. Responders had an average age of 54 years, and this could have influenced the finding with older practitioners having different views from younger ones. Consequently, the average age of practitioners in South Africa is on the increase and 56 years is now considered the average age of a GP working in the country. Although there are many limitations to this study, this is one of the first studies that attempt to examine medical professionals’ views on codeine use in South Africa and adds to the current evidence base. It provides additional information to add to the debate on the availability and sale of OTC codeine medicines.

## Conclusion

The debate on codeine, its availability and indications for use is likely to dominate the discussions surrounding the drug into the future. It is important that policy generation is informed by numerous sources, including doctors, specialists, patients, addiction services and pharmacists. This study aimed to garner information on medical professional perspectives on codeine and has highlighted the concerns of medical professionals in South Africa surrounding codeine use, especially codeine sold OTC. There appears to be a great need to improve education of patients on the consumption of codeine and the risks associated with the drug. Additional screening tools and supports in practice to detect and manage codeine-related issues in primary care require further evaluation. Greater data sources are required to examine the sale and consumption of codeine medicines in the interest of public health.
